# Common Carp Kidney as a Multipurpose Biomarker Organ: Insights from Perfluorooctanoic Acid Exposure

**DOI:** 10.3390/toxics14040287

**Published:** 2026-03-28

**Authors:** Maurizio Manera, Cosma Manera, Luisa Giari

**Affiliations:** 1 Department of Biosciences, Food and Environmental Technologies, University of Teramo, St. Renato Balzarini 1, 64100 Teramo, Italy; 2 Independent Researcher, St. Antonio Di Vincenzo 12/2, 40129 Bologna, Italy; cosma.manera@gmail.com; 3 Department of Environmental and Prevention Sciences, University of Ferrara, St. Luigi Borsari 46, 44121 Ferrara, Italy; luisa.giari@unife.it

**Keywords:** toxicologic pathology, environmental pathology, One Health, translational research, per- and polyfluoroalkyl substances, PFAS

## Abstract

The common carp (*Cyprinus carpio*) kidney uniquely integrates excretory nephrons, renal hematopoietic tissue, and hormonally active thyroid follicles, positioning it as a candidate “multipurpose biomarker organ” for pollutants like perfluorooctanoic acid (PFOA), a prototype long-chain PFAS and persistent organic pollutant exhibiting nephrotoxic, immunotoxic, and thyroid-disrupting effects. Building on prior histological, ultrastructural, and morphometric analyses from carp exposed to waterborne PFOA (0, 200 ng L^−1^, 2 mg L^−1^ for 56 days), a hierarchical multipurpose index comprising nephrotoxic, immunotoxic, and thyrotoxic subindices was developed from z-scored light-, electron-microscopy, and morphometric features, enabling cross-scale integration; proximal tubule vesiculations and effete rodlet cells (RCs) were newly quantified from archival electron micrographs. The subindices captured PFOA-induced glomerular hyperfiltration with proximal protein reabsorption and collecting duct RCs recruitment (nephrotoxic); hematopoietic tissue RCs recruitment, clustering, and exocytosis (immunotoxic); and increased thyroid follicle abundance/vesiculation, cross-sectional area, and perimeter (thyrotoxic). Quantification of previously only qualitatively assessed features provided statistical validation, while radar plot integration rendered results more intuitively evident—particularly highlighting the non-monotonic thyroid response—condensing organ-level complexity into a coherent framework supporting carp kidney as a translational One Health model for multi-endpoint waterborne pollutant assessment.

## 1. Introduction

Fish serve as representative sentinel organisms for biomonitoring waterborne pollutants within a One Health framework, integrating exposure via gills and diet to reflect bioaccumulation, trophic transfer, and ecological risk in a way that directly links environmental contamination to animal and human health [1,2,3,4,5]. Within toxicologic pathology, fish also function as translational bioindicator species because many pathways involved in xenobiotic handling, endocrine regulation, and cytotoxic responses are conserved across vertebrates, despite deep evolutionary divergence [6,7,8]. Among teleosts, the common carp (*Cyprinus carpio*) is particularly suitable for biomonitoring when compared with zebrafish (*Danio rerio*), as it is globally distributed in freshwater systems, easily obtained and maintained in captivity, plays a major ecological and aquaculture role, and constitutes a relevant human food source, thereby bridging environmental pollutants contamination and potential trophic transfer to humans [9,10,11,12,13]. Moreover, the carp’s extended lifetime and ability for bioconcentration and bioaccumulation render it an effective model for evaluating the chronic impacts of persistent pollutants, such as perfluorooctanoic acid (PFOA), surpassing the predominantly laboratory-based representativeness of zebrafish [14,15,16].

**Figure 1:**
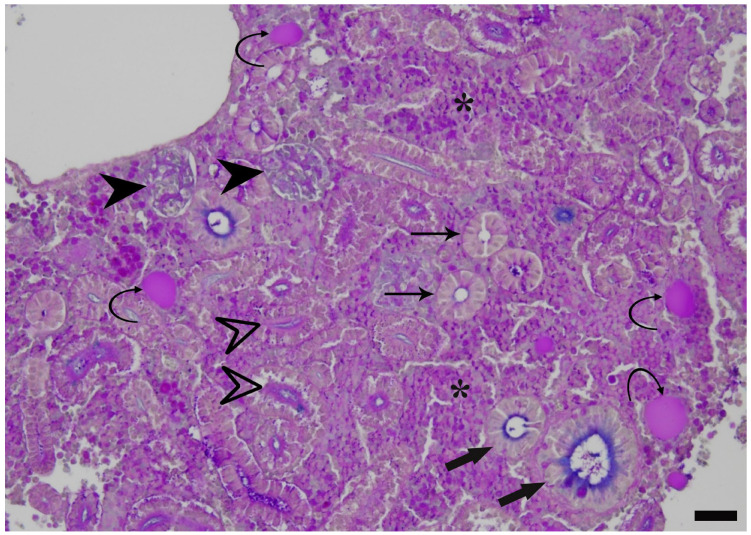
Representative paraffin-embedded tissue section of common carp kidney showing the typical histological pattern. Alcian blue–PAS stain. Scale bar: 25 μm. The following anatomical structures can be observed: nephrons with their main components, renal corpuscles (arrowheads), proximal tubules (empty arrowheads), distal tubules (thin arrows) and collecting ducts (thick arrows); scattered thyroid follicles (curved arrows); and hematopoietic tissue (asterisks).

The common carp kidney (mesonephros) provides a unique anatomical and functional integration of three major compartments: nephrons, which mediate renal excretory function; an extensive hematopoietic interstitium, which sustains erythropoietic and myeloid lineages and thus innate immune surveillance; and hormonally active thyroid follicles (Figure 1), which support endocrine regulation [17,18]. This convergent organisation is not typically combined within a single organ in mammalian models and underpins the proposal of the carp kidney as a “multipurpose biomarker organ” capable of simultaneously capturing nephrotoxic, immunotoxic, and thyrotoxic effects under realistic exposure scenarios [19]. Subchronic waterborne PFOA exposure (0, 200 ng L^−1^, 2 mg L^−1^ for 56 days) in carp has already been shown to induce: (i) nephrotoxic changes, including dilation of the glomerular capillary bed, enhanced proximal tubule vesiculation, and collecting duct rodlet cells (RCs) recruitment; (ii) immunotoxic responses, particularly recruitment, clustering, and exocytosis of RCs in the renal hematopoietic tissue; and (iii) thyrotoxic effects, such as increased abundance of renal thyroid follicles and enhanced vesiculation of colloid and follicular epithelium, with morphologic alterations occurring even when tissue PFOA concentrations were below the analytical detection limit [19,20,21,22,23]. Though RCs’ putative immune function rests on indirect evidence and remains a widely shared working hypothesis, they are currently regarded as teleost-specific immune cells whose definitive role is yet to be conclusively established [24,25,26].

PFOA is a prominent long-chain per- and polyfluoroalkyl substance (PFAS), often utilised as a prototype due to its comprehensive characterisation regarding environmental persistence, toxicokinetics, and toxicodynamics. PFOA and related long-chain perfluorocarboxylic acids have been listed under the Stockholm Convention as persistent organic pollutants (POPs), reflecting their resistance to degradation, global distribution, and bioaccumulation potential in wildlife and humans [27,28,29]. Experimental and field studies consistently document multi-organ toxicity of PFOA, including hepatotoxic, nephrotoxic, immunotoxic, and endocrine-disrupting effects at environmentally relevant concentrations [1,30,31,32]. Animal and epidemiological data show suppression of antigen-specific antibody responses and broader perturbation of immune function [32,33]. Moreover, PFAS—including PFOA—are increasingly recognised as thyroid disruptors: they can interfere with thyroid hormone homeostasis, modulate thyroid-related gene expression, and exert multi- and trans-generational effects on the thyroid axis [34,35,36].

The integration of heterogeneous biological responses into synthetic metrics has become a pivotal challenge in environmental monitoring, shifting the focus from single-endpoint assessments to integrated organ-level signatures [37]. In aquatic ecotoxicology, several frameworks have been proposed to summarise complex datasets, such as the Integrated Biomarker Response (IBR) index [37] and its subsequent evolutions [38,39,40], which utilise star plots and scoring systems to visualise multi-tissue stress. These integrative approaches are not intended to replace high-resolution individual pathology, but rather to act as a multiparametric bridge, condensing cross-scale information—from ultrastructural alterations to histomorphometric shifts—into a coherent diagnostic profile [41]. However, while composite indicators are widely applied to diverse organs (e.g., liver or gills), their application to multi-functional organs remains largely unexplored [42,43,44]. Building upon this methodological tradition, the present study proposes a modular, hypothesis-driven framework to characterise the common carp kidney as a multipurpose biomarker organ, utilising z-scoring and hierarchical subindex integration to capture the synergistic effects of waterborne pollutants.

In more detail, the aim of the present research was to reassess, in an integrative way, previous quantitative and qualitative data [19,20,21,22,23] on PFOA-induced alterations in these three renal compartments—nephron (renal), hematopoietic (immune), and thyroid follicles (endocrine)—in order to consolidate the concept of the carp kidney as a candidate multipurpose biomarker organ [19]. To this end, a hierarchical multipurpose index was developed, consisting of three subindices (nephrotoxic, immunotoxic, and thyrotoxic), conceived as a modular, hypothesis-driven strategy in which individual features can be selected and weighted according to the expected endpoint, thereby maximising biological relevance and minimising redundant measurements rather than pursuing a fixed “metric purpose” akin to a standard reference scale. Z-scoring was applied to a curated subset of histological, ultrastructural and morphometric features previously demonstrated to be responsive to PFOA in the same experimental fish cohort, ensuring comparability across scales and tissues. For two key features—“proximal tubule vesiculations” and the occurrence of “effete RCs”—quantitative scoring was performed de novo in the present study using archival transmission electron micrographs, thus expanding the available dataset while maintaining methodological continuity with earlier work on this cohort.

## 2. Materials and Methods

The present research relied entirely on statistical re-elaboration of quantitative data from previously published articles on the same PFOA-exposed carp experimental cohort or on original quantification of previously qualitatively described results [19,20,21,22,23,45]. The original experiment [46] consisted of 3 groups of common carp, *C. carpio*: unexposed, 200 ng L^−1^ PFOA and 2 mg L^−1^ PFOA for 56 days under sub-chronic conditions in a continuous flow-through system. For details of the original experiment, readers are referred to Giari et al. (2016) [46], from which all subsequent pertinent studies derive. This approach ensured full homogeneity across datasets from identical fish samples, avoiding the need to sacrifice additional animals.

### 2.1. Feature Selection and Elaboration from Previous Research

Relevant quantitative and qualitative features—specifically, light- and electron-microscopy and morphometric characteristics showing distinctive alterations among experimental groups—were preliminarily selected from previous research as reported in Table 1. Notably, the lack of statistical evaluation of prior qualitative findings represented a possible limitation that the present study addresses through re-analysis and original quantification. Features were grouped according to their pathophysiological impact into nephrotoxic, thyrotoxic, and immunotoxic features (Table 1). For the nephrotoxic subindex, features describing glomerular filtration barrier integrity were included (podocyte pedicel width, thickness of the glomerular basement membrane, and width of endothelial fenestrae in glomerular capillaries) [47,48,49], together with tubular cytological alterations (degree of proximal tubule cell vesiculation and occurrence of RCs in collecting ducts) [23,50,51]. For the thyrotoxic subindex, morphological and morphometric features of thyroid follicles activity were used (total number of renal thyroid follicles, number of follicles displaying consistent vesiculations at the colloid–epithelium interface, and thyroid follicle cross-sectional area and perimeter) [19,52,53,54]. For the immunotoxic subindex, ultrastructural and morphometric features reflecting RCs recruitment and activation were considered (number of RCs in the renal hematopoietic tissue, proportion of effete RCs showing complete expulsion of cytoplasmic content, and RC degranulation activity) [24,26,55].

For certain features—specifically “proximal tubule vesiculations” and “effete RCs”—quantification was performed de novo for this study using archival ultrastructural images from ultrathin sections (TEM grids). These images were systematically captured during previous studies (Table 1) from kidney samples of 5 fish per experimental group. The feature “RC degranulation” was obtained as the mean value of the factor score from linear discriminant analysis from a previous statistical dataset (Table 1).

Proximal tubule vesiculations were quantified via image analysis of proximal tubule cell regions of interest (ROIs). The ratio of vesiculated cytoplasm area to total cytoplasmic area was calculated using Orbit Image Analysis software (version 3.64, Actelion Pharmaceuticals Ltd., Allschwil, Switzerland; GPLv3 licence) [56]. Object segmentation followed the manufacturer’s protocols [56]: two classes were defined (vesiculated cytoplasm and background cytoplasm), trained via manual outlining on representative images from all experimental groups, then applied across all images with parameter refinements via the feature configuration interface as needed.

The proportion of effete RCs was determined by counting effete RCs relative to all RCs documented in ultrastructural micrographs.

### 2.2. Subindices and Multipurpose Index: An Exploratory Analytical Framework

**Figure 2:**
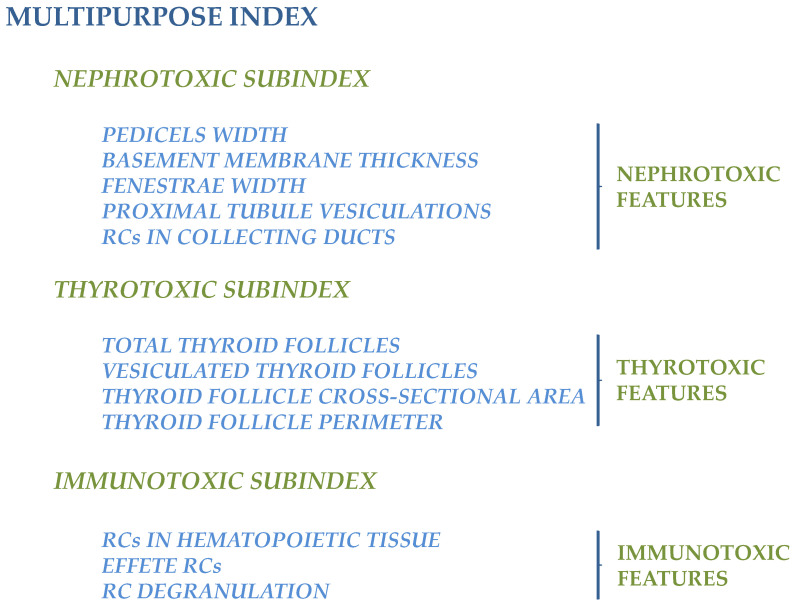
Schematic hierarchical representation of the multipurpose index, composed of nephrotoxic, thyrotoxic, and immunotoxic subindices, where individual features serve as replicates within each subindex.

Each feature was introduced as a replicate to compose three subindices, according to its pathophysiological impact: nephrotoxic, thyrotoxic, and immunotoxic. Cumulatively, all features contribute to the definition of the multipurpose index, as schematically depicted in Figure 2.

In more detail, the development of the nephrotoxic, thyrotoxic, and immunotoxic subindices, culminating in the overall multipurpose index, is conceived as an exploratory, hypothesis-driven analytical framework rather than a finalised, reference-scale metric. The primary objective of this work was to provide a proof-of-concept for the carp kidney as an integrated, multi-compartment biomarker system. Crucially, this higher-level synthesis is strictly complementary and does not seek to replace traditional inferential statistics based on individual fish variability. All foundational biological effects and inter-individual variances were rigorously established at the individual level in prior studies [19,20,21,23,45], which served as the essential quality control and “ground truth” for the current integration. Within this data flow, using multiple features within each subindex is a deliberate choice for multibiomarker characterisation (e.g., Integrated Biomarker Response approach [37]), aimed at revealing emergent organ-level patterns—such as the non-monotonic thyroid response—that may remain latent in single-trait analyses.

### 2.3. Statistical Analysis

Individual features were z-scored by averaging across experimental groups for each feature. For example, pedicel width values were averaged across the three experimental groups (unexposed: 228 nm; 200 ng L^−1^ PFOA: 228 nm; 2 mg L^−1^ PFOA: 286 nm) to obtain a mean (μ = 247.33 nm) and respective standard deviation (σ = 33.49 nm). Each group’s value was then z-scored using the following formula: z = (X − μ)/σ, where X is the group representative value, μ is the mean across all three experimental groups for that feature, and σ is the respective standard deviation across all three experimental groups. This produces dimensionless z-scores for each group (e.g., pedicel width: unexposed: −0.58; 200 ng L^−1^ PFOA: −0.58; 2 mg L^−1^ PFOA: +1.15), which were then cumulated within each subindex and finally within the multipurpose index as replicates. Original features data (scale measures, counts, and ratios) vary dramatically in absolute magnitude and biological scale. Z-score transformation places all endpoints on a common statistical scale while preserving relative deviations from means, facilitating synthesis of complex multiparametric data into coherent toxicity indices without arbitrary weighting [41].

In line with this exploratory scope, equal weighting was assigned to all z-scored features. This approach is intentionally conservative: it avoids a priori bias regarding the relative functional “importance” of heterogeneous endpoints (e.g., ultrastructural vs. histomorphometric features), which may vary depending on the specific pollutant, and is consistent with guidance on composite indicators [57]. As this is a modular proposal, researchers are encouraged to tailor feature selection and weighting according to their specific experimental endpoints [57]. By maintaining a transparent and non-substitutive approach, the z-score transformation harmonises diverse scales into an intuitive composite profile (radar plots), highlighting the potential of the carp kidney as a translational One Health model for aquatic pollution and capturing non-monotonic endocrine disruptor-like responses [58].

Thereafter, since data were not normally distributed, the multipurpose index and each subindex separately were tested for significant differences among experimental groups using the Kruskal–Wallis test followed by Dunn’s pairwise comparisons (p Holm-corrected) in JASP (version 0.19.5.4; JASP Team, Amsterdam, The Netherlands). Given the sample characteristics, Monte Carlo significance estimation was also applied to the Kruskal–Wallis test for more conservative and precise *p*-value estimation, using SPSS^®^ (version 14.0.2, SPSS Inc., Chicago, IL, USA). For rapid, intuitive evaluation of z-scored data among experimental groups, radar plots were generated.

## 3. Results

**Figure 3:**
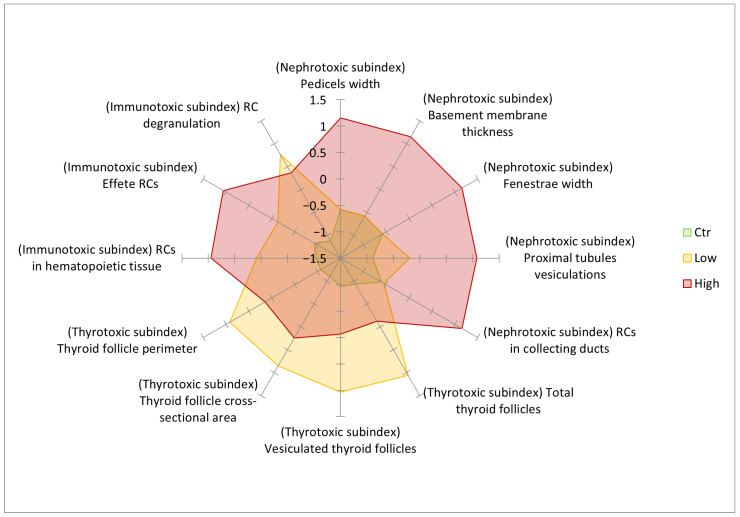
Radar plot of z-scored feature values for each experimental group (unexposed, 200 ng L^−1^ PFOA, 2 mg L^−1^ PFOA), organised by subindex (nephrotoxic, thyrotoxic, and immunotoxic). The thyrotoxic subindex shows a non-monotonic dose–response: only the 200 ng L^−1^ PFOA group differs significantly from unexposed controls, while the 2 mg L^−1^ PFOA group does not differ significantly from either (cf. Table 3).

Results are reported in Table 2 and Table 3 and Figure 3. Monte Carlo significance estimation provided more conservative and precise *p*-values compared to the asymptotic method for all subindices: nephrotoxic subindex (*p* < 0.001 vs. asymptotic *p* = 0.003), thyrotoxic subindex (*p* < 0.001 vs. asymptotic *p* = 0.007), and immunotoxic subindex (*p* = 0.012 vs. asymptotic *p* = 0.039). The thyrotoxic subindex revealed a large effect size (Table 2), with highly significant non-monotonic dose–response and correct rank-biserial correlations (r_rb_) confirming a U-shaped dose–response pattern (Table 2 and Table 3; Figure 3).

Effect sizes (ε^2^ = 0.811–0.895, Table 2) confirmed large biological impacts across all subindices. Pairwise comparisons (Table 3) showed r_rb_ values approaching ±1.0 for key contrasts (unexposed vs. exposed), demonstrating robust group separation. Critically, only the multiparametric index detected significant differences between unexposed controls and both exposure levels (*p* < 0.01, r_rb_ = −0.938 to −1.000), while individual subindices missed at least one contrast. These three distinct patterns—high-dose kidney damage, thyroid peak-then-drop, and high-dose immune activation—integrate synergistically in the multiparametric index for superior group separation. Figure 3 graphically highlights these patterns across subindices in an intuitive, aggregated format, particularly the thyrotoxic subindex’s non-monotonic dose–response and RC degranulation peak (immunotoxic feature), making these trends immediately visually apparent.

## 4. Discussion

The present study confirms and extends previous evidence that the carp kidney represents a systematic, integrated multipurpose biomarker organ capable of capturing nephrotoxic, immunotoxic, and thyrotoxic effects of PFOA within a single anatomical district [19,20,21,22,23,45]. By extracting and z-scoring morphometric, histological, and ultrastructural endpoints previously described separately for the nephron, renal hematopoietic tissue, and thyroid follicles [19,20,21,22,23,45], it was possible to derive composite subindices that synthesise tissue-specific toxicity, culminating in the multipurpose index as their integrated synthesis. This approach should not be interpreted as pursuing a “metric purpose,” namely the development of a reference/standard metric, but rather as a modular, hypothesis-driven strategy where features can be selected and weighted according to the expected endpoint, maximising biological relevance while minimising redundant measurements [59,60].

The nephrotoxic subindex—derived from features such as pedicel width, basement membrane thickness, fenestrae alterations, proximal tubule vesiculations, and RCs increases in renal collecting ducts as a result of their affection—recapitulates the ultrastructural picture of glomerulonephrosis and related tubular involvement [48,50,51,61] previously described in the same fish [22,23]. At the highest exposure concentration (2 mg L^−1^ PFOA), podocyte effacement occurred with widening/disarrangement/fusion of pedicels into continuous cytoplasmic sheets (average width 286 nm), drastic reduction/loss of filtration slits and slit diaphragms, basement membrane disarrangement/enlargement (average thickness 237 nm), slightly enlarged endothelial fenestrae (average width 180 nm) with irregular villous-like projections, focal podocyte vacuolations, and foamy proteinaceous material in the urinary space, together with enhanced endocytic activity (increased/large flocculent vesiculations) in the first proximal tubule segment [22,23]. These lesions align with the broader literature on podocyte injury and proteinuric kidney disease, where foot process effacement and slit diaphragm loss represent key structural correlates of barrier failure [62,63], potentially involving organic anion transporters (OATs) in podocytes and proximal tubules that facilitate PFOA renal handling [22,23,64,65]. The strongly positive z-scores for all nephrotoxic features at 2 mg L^−1^ PFOA quantify the uniformity and magnitude of damage across glomerular and tubular compartments, consistent with this morphopathological pattern [22,23]. Conversely, the mildly negative to near-zero values in the unexposed and low- concentration (200 ng L^−1^ PFOA) groups reflect baseline variability or only incipient, sub-threshold lesions—previously reported as glomerular capillary bed dilation with loss of folding pattern, and early proximal tubular vesiculation—reinforcing nephron sensitivity to high experimental PFOA concentrations, while environmentally relevant levels produce only early alterations detectable at ultrastructural resolution [22,23].

In the present study, recoding the previously published morphometric, histological, and ultrastructural endpoints [19,20] into a composite thyrotoxic subindex provides clearer visualisation of PFOA effects on the carp thyroid and, in particular, made the non-monotonic nature of the response more immediately appreciable. In earlier work on the same experimental cohort [19,20], the environmentally relevant concentration of 200 ng L^−1^ PFOA was already shown to induce marked thyroid folliculogenesis, with a high number of small, morphologically altered follicles and a pronounced reduction in colloid area and perimeter, whereas fish exposed to 2 mg L^−1^ PFOA exhibited relatively larger follicles but more evident degenerative ultrastructural changes, including rough endoplasmic reticulum enlargement, cytoplasmic vacuolation, and features consistent with early degeneration. These findings were interpreted as evidence that 200 ng L^−1^ PFOA primarily acts as an endocrine disruptor and goitrogen, driving TSH-mediated follicle recruitment and colloid depletion, while the higher dose superimposes a more frankly thyrotoxic and cytotoxic component on thyroid tissue, in line with concurrent alterations in liver and kidney observed in the same fish [19,20] and with the broader literature on PFAS-induced thyroid disruption in fish and other vertebrates [34,66,67]. By collapsing these complex and partially divergent endpoints into a single thyrotoxic subindex, the current analysis highlights a non-monotonic dose–response in which the 200 ng L^−1^ PFOA group is coherent with an endocrine-disruptor-like signature and the 2 mg L^−1^ PFOA group is in accordance with a more toxic/degenerative profile, a pattern that is consistent with the conceptual framework of endocrine disruptors and with the widespread occurrence of non-monotonic dose–responses described for hormones and endocrine-disrupting chemicals [58,68].

The immunotoxic subindex, based on RCs occurrence in hematopoietic tissue, effete RCs, and RC degranulation, synthesises the immunotoxic and innate immune activation profile previously documented in detail in the same fish [21,23,45]. RCs have been proposed as sentinel cells of chemical and parasitic stress in fish, with roles linked to innate immunity and inflammatory responses [24,55,69,70]. In carp exposed to PFOA, earlier work demonstrated increased numbers of RCs in the renal hematopoietic interstitium, clustering, closer association with myeloid cells, and ultrastructural evidence of intense exocytosis and degeneration, particularly at 2 mg L^−1^ PFOA, but already evident at 200 ng L^−1^ PFOA [21,23,45]. The z-scored immunotoxic features show a consistent shift from negative values in controls to positive values in exposed groups, indicating a dose-related activation of RCs recruitment and turnover, with degranulation objectively quantified via texture analysis/LDA [45]. Of note, RC degranulation peaks at the lower concentration, suggesting that environmentally relevant exposure can elicit a vigorous secretory response [21]. This non-linear nuance is highlighted in the z-scores and underscores the high sensitivity of rodlet cells to low-level PFOA stress [21,23,24,45]. The possible role of PFAS in disturbing teleost immune function, including specialised cell types such as RCs, has recently been reviewed by Cai et al. (2026) [71], who largely build on the limited experimental evidence currently available for RCs responses to PFOA in carp kidney [21,23]; as a result, PFAS–RC interactions remain only sparsely explored and mostly discussed at a conceptual level. Growing experimental and observational data support the view that RCs act as stress-responsive innate immune effector or sentinel cells in teleosts [24,25,70,72], while PFAS are increasingly recognised as modulators of innate immune pathways, including TLR–NF-κB signalling, in fish and other vertebrate immune cells [30,73,74]. Against this broader mechanistic background, the PFOA-induced changes in RCs recruitment and degranulation observed in the present cohort are consistent with the hypothesis that PFAS may influence RC activity indirectly, via upstream activation of classical innate sentinel cells and cytokine networks, or potentially through pattern-recognition pathways analogous to TLR-driven NF-κB activation; however, such receptor-level mechanisms remain to be demonstrated directly in RCs [21,23]. Together, these findings support the use of RC-based metrics as a component of an immunotoxic index and provide quantitative backing to the proposal that RCs function as biomarkers of PFAS exposure in fish, complementing classical leukocyte or cytokine endpoints [21,23,24,45].

With regard to possible limitations and biases of the adopted methods—particularly z-scoring—it should be stressed that, although this permits direct comparison of heterogeneous measures, several limitations must be acknowledged [41,75]. First, the indices derive from previously published datasets not originally designed for integrative scoring; hence, equal weighting of individual features may not reflect their relative functional importance [57,76]. Second, collapsing complex biological phenomena into single indices can mask divergent endpoint behaviours, such as the non-monotonic dose–response of the thyrotoxic subindex or the peak RC degranulation at low concentration [77,78]. Third, while z-scores highlight relative deviations within the studied cohort, they remain inherently context-dependent and should not be overinterpreted as absolute toxicity thresholds applicable across species or exposure scenarios [79,80]. Despite these caveats, the consistency between composite subindices and the detailed histopathological, ultrastructural, and morphometric narratives of the original articles [19,20,21,22,23,45] demonstrates the biological meaningfulness of the z-scored approach. It successfully translates rich qualitative descriptions of PFOA-induced kidney pathology into quantitative metrics comparable across tissues, doses, and potentially contaminants. In other words, the proposed methodology—grounded in the quantitative statistical elaboration of original qualitative and quantitative data from prior histological, morphometric, and ultrastructural research documenting nephrotoxicity, immunotoxicity, and thyrotoxicity separately in carp kidney [19,20,21,22,23,45]—represents a complementary extension to these traditional approaches rather than an alternative, intuitively summarising and integrating such data into immediately appreciable quantitative metrics.

Taken together, the integrated analysis confirms that PFOA exerts multi-compartment toxicity in the carp kidney, spanning the glomerular filtration barrier and tubular integrity [22,23], innate immune involvement through rodlet cells [21,23], and thyroid follicle structure and colloid dynamics [19,20]. The dose-dependent and, in some cases, non-monotonic patterns highlight the importance of considering both environmentally relevant exposures and higher experimental doses when characterising PFAS hazards [23,34,58].

Building on these confirmed PFOA effects, the carp kidney offers a parsimonious model for integrated One Health assessment, because it uniquely hosts three major functional compartments—nephrons, renal hematopoietic tissue, and hormonally active thyroid follicles—that in mammals are distributed across distinct organs (kidney, bone marrow, lymphatic organs, and thyroid gland) [17,81]. The One Health paradigm specifically emphasises the interconnectedness of environmental quality, animal health, and human well-being in the context of waterborne pollutants [82,83,84], making the carp kidney’s integrated pathology an ideal translational bridge across these domains. This organisation permits simultaneous evaluation of nephrotoxic, immunotoxic, and thyrotoxic effects on a single histological slide or electron microscopy grid from the same individual, reducing animal use and enabling truly multiparametric interpretation under identical exposure conditions. In the present work, PFOA was used primarily as a case-study pollutant to demonstrate the feasibility and interpretive value of this integrated, index-based approach; however, the intention is that the same multiparametric framework could be applied more broadly to other waterborne contaminants, with the carp kidney being considered and further developed as a multipurpose biomarker organ. Previous studies on this experimental cohort have shown that PFOA can induce clear ultrastructural and morphometric alterations in the different kidney structures even when tissue PFOA concentrations fall below analytical detection limits, underscoring that biologically relevant effects may occur at, or below, the sensitivity threshold of routine chemical assays [19,22,23]. In such scenarios, integrated morphologic and index-based approaches may provide a valuable complementary tool for environmental monitoring, especially for widely farmed and consumed species such as common carp, which link aquatic contamination, trophic transfer, and human dietary exposure to PFAS and other waterborne pollutants [11,14,85,86]. Encouraging the use of histopathology-, ultrastructure-, and morphometry-derived indices alongside chemical analyses is therefore consistent with the One Health paradigm, as it captures converging evidence on renal, immune, and thyroid disruption that is relevant for ecosystem health, animal welfare, and potential human health risks from chronic PFAS exposure.

The carp kidney emerges as a compact, information-rich target for histopathological, morphometric, and ultrastructural biomarker development, and the index-based framework proposed here offers a practical way to synthesise complex morphological data. Future work could expand this strategy by incorporating additional endpoints (e.g., molecular markers, oxidative stress indices [87]) or by testing other PFAS and contaminant mixtures, thereby refining the use of carp and its kidney as a multipurpose biomarker system for aquatic pollution evaluation and for comparative toxicological research in a One Health and translational perspective.

## 5. Conclusions

Z-scoring of heterogeneous morphometric, histological, and ultrastructural endpoints from prior studies enabled their direct comparison both within individual subindices (thyrotoxic, nephrotoxic, and immunotoxic) and across the composite multipurpose index, providing a more comprehensive and coherent synthesis of carp kidney responses to PFOA that transcends the singular analyses previously published. Qualitative observations originally described in earlier work—such as proximal tubule cell vesiculations and effete RCs—were here quantitatively codified for the first time, while preexisting measurements (e.g., glomerular filtration barrier alterations) were integrated into cumulative statistical assessments within the respective subindices. This approach culminates in the multipurpose index as an integrative synthesis of its constituent subindices, positioning the carp kidney as a versatile biomarker organ for multiparametric toxicological evaluation in the One Health paradigm and translational research context, linking aquatic contamination through trophic transfer in this widely consumed food fish to potential human renal, immune, and thyroid health risks, and underscoring the value of morphology-based indices as complementary tools to chemical analysis in PFAS monitoring. While this study employed PFOA solely as a case study to provide insights into the common carp kidney as a candidate multipurpose biomarker organ, toxicologic pathology researchers are encouraged to tailor feature selection and weighting according to their specific endpoints of interest; although the multipurpose index was not developed as a standalone metric, this potential merits consideration pending further validation and research.

## Figures and Tables

**Table 1:** Original (i.e., before z-scoring) feature values according to each subindex from prior research (publications, archival materials, and datasets) for the three experimental groups (unexposed, 200 ng L^−1^ PFOA, 2 mg L^−1^ PFOA).

Subindex	Feature	Experimental Groups			Note	Reference
		Unexposed	200 ng L^−1^ PFOA	2 mg L^−1^ PFOA		
Nephrotoxic	Pedicels width (nm)	228	228	286	Authors reported that only 2 mg L^−1^ PFOA affected the glomerular filtration barrier; therefore, the measurement from fish exposed to 200 ng L^−1^ PFOA was assumed to be equivalent to that of unexposed fish in the current study	[22]
	Basement membrane thickness (nm)	146	146	237	Authors reported that only 2 mg L^−1^ PFOA affected the glomerular filtration barrier; therefore, the measurement from fish exposed to 200 ng L^−1^ PFOA was assumed to be equivalent to that of unexposed fish in the current study	[22]
	Fenestrae width (nm)	136	136	180	Authors reported that only 2 mg L^−1^ PFOA affected the glomerular filtration barrier; therefore, the measurement from fish exposed to 200 ng L^−1^ PFOA was assumed to be equivalent to that of unexposed fish in the current study	[22]
	Proximal tubule vesiculations	0.0357	0.0557	0.0918	Vesiculated area/total cell area, quantified from representative archival ultrastructural images for the current study	[22]
	Rodlet cells (RCs) in collecting ducts	0	0.01	0.35	Mean number of RCs per microscopic field (semithin epoxy resin-embedded sections), evaluated for the current study from the dataset of previous research	[23]
Thyrotoxic	Total thyroid follicles	5.00	42.33	20.29	Mean total number of follicles per histological section (paraffin-embedded)	[19]
	Vesiculated thyroid follicles	1.33	28.50	13.57	Mean total number of follicles per histological section (paraffin-embedded)	[19]
	Cross-sectional area (µm^2^)	829	295	461	Median values	[20]
	Perimeter (µm)	113	69	86	Median values	[20]
Immunotoxic	RCs in hematopoietic tissue	0.81	4.02	6.47	Mean number of RCs per microscopic field (semithin epoxy resin-embedded sections)	[23]
	Effete RCs	0.1	0.2	0.35	Effete RCs/total RCs, quantified from representative archival ultrastructural images for the current study	[21]
	RC degranulation	−0.98438	0.292708	0.022556	Factor score (mean value) from linear discriminant analysis, evaluated for the current study from the dataset of previous research	[45]

**Table 2:** Kruskal–Wallis test.

Index/Subindex	H Statistic	df	*p*-Value	ε^2^ (Rank)	95% CI for ε^2^
Multipurpose	25.12	2	<0.001	0.718	[0.643, 0.828]
Nephrotoxic	11.450	2	0.003	0.818	[0.815, 0.959]
Thyrotoxic	9.846	2	0.007	0.895	[0.901, 0.931]
Immunotoxic	6.489	2	0.039	0.811	[0.695, 0.973]

**Table 3:** Dunn’s pairwise comparisons (P_Holm_ corrected) with correct rank-biserial correlation signs (r_rb_).

Index/Subindex	Comparison	z	r_rb_	P_Holm_
Multipurpose	Unexposed − 2 mg L^−1^ PFOA	−4.943	−1.000	<0.001
Multipurpose	Unexposed − 200 ng L^−1^ PFOA	−3.185	−0.938	0.003
Multipurpose	2 mg L^−1^ PFOA − 200 ng L^−1^ PFOA	+1.758	+0.597	0.079
Nephrotoxic	Unexposed − 2 mg L^−1^ PFOA	−3.336	−1.000	0.003
Nephrotoxic	Unexposed − 200 ng L^−1^ PFOA	−1.173	−0.640	0.241
Nephrotoxic	2 mg L^−1^ PFOA − 200 ng L^−1^ PFOA	+2.163	+1.000	0.061
Thyrotoxic	Unexposed − 2 mg L^−1^ PFOA	−1.569	−1.000	0.233
Thyrotoxic	Unexposed − 200 ng L^−1^ PFOA	−3.138	−1.000	0.005
Thyrotoxic	2 mg L^−1^ PFOA − 200 ng L^−1^ PFOA	−1.569	−1.000	0.233
Immunotoxic	Unexposed − 2 mg L^−1^ PFOA	−2.534	−1.000	0.034
Immunotoxic	Unexposed − 200 ng L^−1^ PFOA	−1.491	−1.000	0.272
Immunotoxic	2 mg L^−1^ PFOA − 200 ng L^−1^ PFOA	+1.043	+0.778	0.297

## Data Availability

The raw data supporting the conclusions of this article will be made available by the authors on request.
